# The association between school bullying involvement and Internet addiction among Chinese Southeastern adolescents: a moderated mediation model with depression and smoking

**DOI:** 10.3389/fpsyt.2025.1557108

**Published:** 2025-03-25

**Authors:** Yuhang She, Liping Li

**Affiliations:** ^1^ School of Public Health, Shantou University, Shantou, China; ^2^ Injury Prevention Research Center, Shantou University Medical College, Shantou, China

**Keywords:** school bullying, internet addiction, depression, smoking, adolescents

## Abstract

**Background:**

School bullying and Internet addiction are both common public health problems for adolescents. Several studies found an association between school bullying and Internet addiction; however, the underlying mediating and moderating mechanisms of the complex relationship between school bullying and Internet addiction are limited.

**Objective:**

This study explored the mediating role of depression in the relationship between school bullying and Internet addiction and whether smoking moderated the relationship between school bullying and depression in Chinese southeastern adolescents.

**Methods:**

A cross-sectional study was conducted in Guangdong Province in Southeast China in June 2021. Associations between Internet addiction, school bullying, and depression were estimated using Spearman correlation analysis, the mediation effect and moderation effect were examined using Model 4 and Model 7 in the Hayes’ PROCESS macro.

**Results:**

The results included 1992 adolescents, 23.5% and 28.0% of participants reported experiences of school bullying and Internet addiction, respectively. There was a significant correlation between school bullying, depression, and Internet addiction (*p* < 0.01). School bullying direct effects on Internet addiction [*β* = 0.565, *SE*= 0.053, 95% *CI* (0.461, 0.669)], depression partially mediated the association between school bullying and Internet addiction, with the mediation effect size being 36.5%. And smoking played a moderating role between school bullying and depression [*β* = -0.166, *SE*= 0.058, 95% *CI* (-0.280, -0.052)].

**Conclusions:**

In Chinese southeastern adolescents, depression mediated the relationship between school bullying and Internet addiction, and smoking moderated the relationship between school bullying and depression.

## 1 Introduction

School bullying is a common public health problem across the world and has significant impacts on children and adolescents’ physical and mental health, including anxiety, self-harm, or even suicide (1–3). The United Nations Educational, Scientific and Cultural Organization (UNESCO) recently defined school bullying as a damaging social process that is characterized by an imbalance of power driven by social and institutional norms, which is often repeated and manifests as unwanted interpersonal behavior among students or school personnel that causes physical, social, and emotional harm to the targeted individuals or groups, and the wider school community (4). Specifically, school bullying could be categorized into physical, verbal, relational, and cyber bullying, and each type of school bullying involvement could be classified as bullying victimization, bullying perpetration, or bullying perpetration-victimization (3, 5). According to the Health Behavior in School-aged Children (HBSC) study from the World Health Organization (WHO), 29.8% of adolescents have experienced school bullying behaviors globally (6, 7). In China, it is discovered that the prevalence of school bullying ranges from 8.03% to 47.35% among adolescents (8–10). Furthermore, school bullying involvement of adolescents is correlated with substance use (e.g. cigarette smoking and alcohol drinking) and Internet use (9, 11, 12).

The widespread usage of smartphones has inevitably increased the Internet usage in China as well as in the world (13). Internet addiction, as a new emerging clinical disorder, is defined as an impulse-control disorder that does not involve an intoxicant (14). Studies have shown that Internet addiction is a widespread phenomenon among individuals, from adolescents to adults or even older people, and it may cause a series of side effects on people’s health, such as stress, anxiety, and poor sleep quality (15, 16). The global prevalence of Internet addiction in youth ranges from 9% to 52% (17–19), with similar rates (9.3% to 27%) reported in China (20–22). It was suggested that the level of Internet addiction is directly positively associated with youth bullying behaviors (11). For instance, a Spanish longitudinal study found that cyberbullying victimization predicted problematic Internet use (23), while a German longitudinal study concluded that the emergence of traditional and cybervictimization predict problematic Internet gaming behaviors among adolescents, with traditional victimized boys showing higher likelihood of such behaviors than girls (12).

The prevalence of depression among Chinese adolescents has increased over the past decades, it was estimated that the prevalence of depression among Chinese adolescents increased to 26.17%, while adolescents who had experienced school bullying had a higher risk of developing depression (24, 25). A Turkish school-based cross-sectional study with a sample of 6202 middle and high school students revealed that all forms of school bullying involvement (perpetrators, victims, or both victim-perpetrators) are significantly associated with mental health problems, like depression (3). Additionally, a longitudinal study in Canada further confirmed that bullying victimization correlates with depressive symptoms at every time point over a 5-year follow-up (26). Meanwhile, it is reported that adolescents with depressive symptoms were related to receive less social support, which increased chance of being bullied among peers, which supports the bidirectional relationship between depression and bullying victimization (27).

Similarly, numerous studies link Internet addiction and depression in youth (16, 17). A cross-sectional study of Egyptian university students found a strong correlation between Internet addition and depression (28). Moreover, the bidirectional effects between Internet addiction and depression among adolescents were demonstrated in a Chinese longitudinal study among 12043 undergraduates, with the stability of both conditions over a 12-month time span indicating their persistent association (29). Therefore, this findings suggest that depression may mediate the relationship between Internet addiction and school bullying. While studies have explored depression’s mediating role in Internet addiction and either bullying victimization or cybervictimization (30, 31), evidence on its mediation across broader types of school bullying behaviors remains limited.

According to the Global Youth Tobacco Survey, cigarette smoking rates among adolescents are 11.3% in boys and 6.1% in girls, making youth smoking a worldwide public health concerns (32). It is undeniable that cigarette smoking is a major risk factor for interpersonal bullying behaviors and psychological disorders among adolescents (33, 34). A recent Chinese cross-sectional study of 35893 adolescents indicated that high depressive risk, bullying perpetrators, bullying victims, and perpetrator-victims were positively associated with current cigarette smoking habits (35). Interaction between smoking and Internet addiction in neurological mechanisms has also been demonstrated previously (36). However, little is known about the moderating role of adolescents’ cigarette smoking in the relationship between school bullying, depression, and Internet addiction. A study examining whether smoking behaviors could have a moderating effect on the association of school bullying, Internet addiction, and depression requires further clarification.

**Figure 1:**
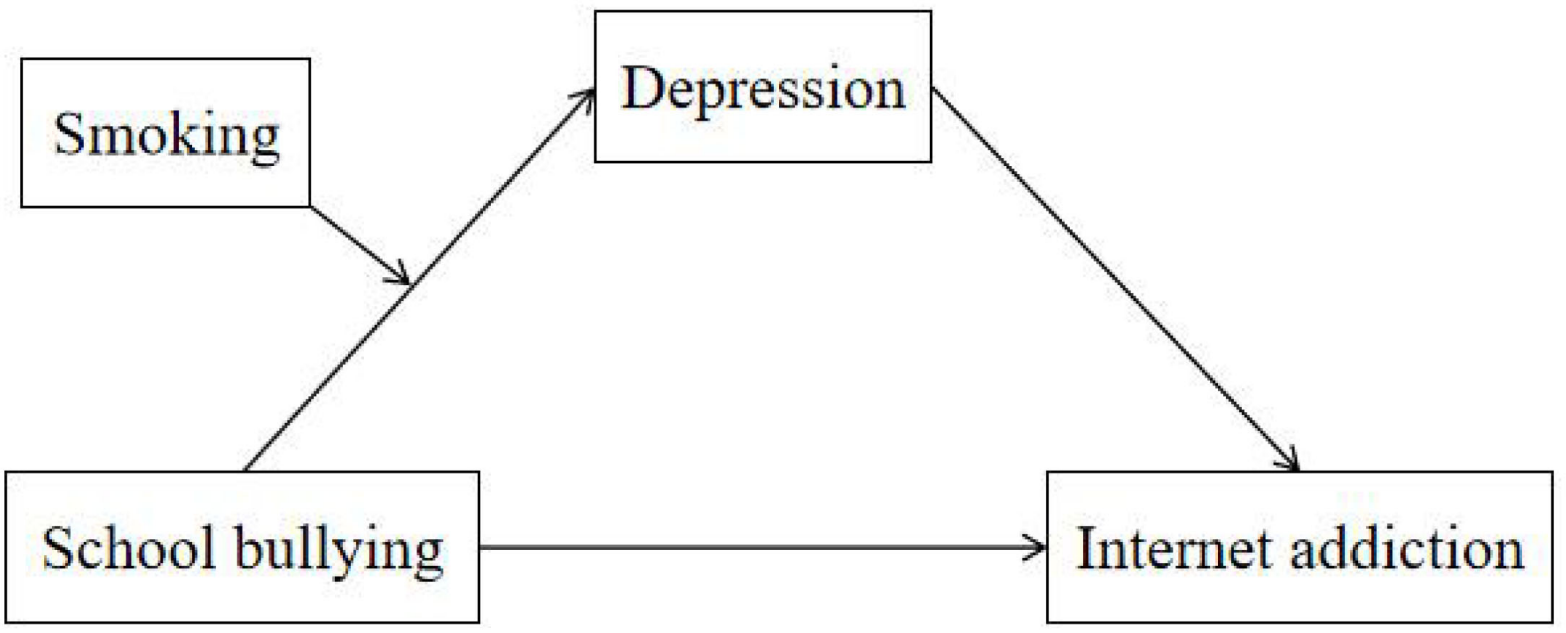
The hypothesized model of the study.

Therefore, we aimed to investigate the mediating role of depression in the association between school bullying and Internet addiction, as well as the moderating role of smoking status on depression in Chinese southeastern adolescents. As shown in **Figure 1**, our study hypothesis are as follows:

Hypothesis 1: School bullying would be positively associated with Internet addiction among adolescents in southeastern China.
Hypothesis 2: Depression would mediate the association between school bullying and Internet addiction in Chinese Southeastern middle school students.
Hypothesis 3: Smoking status would moderate the relationship between school bullying and depression.

## 2 Methods

### 2.1 Participants and procedures

This study used a school-based cross-sectional study data conducted in Shantou, Guangdong Province in Southeast China in June 2021. By using a stratified random cluster sampling method, we first divided Shantou into two areas: downtown and suburban, and participants from 2 middle schools and 2 high schools were finally selected from each area. The self-administered questionnaires were distributed by our trained investigators during the class after introducing the aims of the study and assured the anonymity and confidentiality of the study to the students. Students were allowed to complete the questionnaire within 30 minutes after signing the informed consent. Participants were included if: (a) they were from selected middle schools or high schools, (b) they had obtained written informed consent. And participants were excluded if: (a) they were absent from school during the survey, (b) they were above 18 years old, (c) they had missing data on school bullying, Internet addiction, depression,and smoking status. The Ethics Committee of Shantou University Medical College approved the study.

We distributed 2020 questionnaires to the students, a total of 1992 valid questionnaires were finally included in the study, while 28 unfinished questionnaires or those finished with ≥20% missing data were excluded. In this study, the responding rate was 98.61%.

### 2.2 Measurements

#### 2.2.1 Demographic variables

##### 2.2.1.1 Internet addiction

Internet addiction was assessed by using the Young’s online Internet Addiction Test (IAT) developed by Kimberly S. Young in the US in 1998 (37). The IAT is a 5-point Likert scale with 20-item asking the frequency of each item that aims to identify problematic Internet users: “rarely=1”, “occasionally=2”, “frequently=3”, “often=4”, “always=5”. In the scale, participants were asked to answer questions including salience (eg. “block out disturbing thoughts about life without soothing thoughts of the Internet”), excessive use (eg. “stay online longer than intended”), neglect work (eg. “grades or school work suffer because of the amount of time spend online”), anticipation (eg. “check email before something else that need to do”), lack of control (eg. “others in the life complain about the amount of time spend online”), and neglect social life (eg. “form new relationships with fellow online users”) (37). The total score of the IAT ranges from 20-100, and the higher score indicated the greater level of severity of Internet addiction. In this study, we defined the IAT score ≥50 as Internet addiction, and the IAT score <50 as non-Internet addiction (38, 39). The IAT is a globally used instrument for measuring symptoms of Internet addiction in a wide range of test settings, and has shown a good reliability and validity (40, 41). In this study, the scale’s Cronbach’s alpha was 0.993.

##### 2.2.1.2 School bullying and smoking status

School bullying was measured by using the Juvenile Campus Violence Questionnaire (JCVQ) developed by Chinese scholars (42). The JCVQ is scored by a 4-point Likert scale with 36 items asking the frequency of each event the participant experienced in the past year in school to evaluate physical and psychological school bullying behaviors among Chinese adolescents within 9 dimensions (physical aggression, self-harm, suicide, sexual abuse, verbal aggression, relational aggression, cyber violence, tools violence, and peer pressure): “never=1”, “sometimes=2”, “often =3”, “almost=4” (43, 44). Specifically, for physical school bullying behaviors, participants were asked by the items such as: “I have been hit, kicked, pushed or shoved”, “I’ve been in gang fights”, “I’ve hurt classmates with knives or sticks”, “I’ve been robbed”, etc. Psychological school bullying behaviors contain items “I have been made fun of”, “I’ve been verbally threatened”, “I’ve disclosed other people’s personal information online”, etc. Any item of the JCVQ with a score > 2 indicates the participants involved in school violence behaviors. The JCVQ has shown good reliability and validity (43). In this study, the Cronbach’s alpha was 0.954.

Smoking status was evaluated by a single question: “How often do you smoke?” The value of smoking status was 1 when never smoking was answered, 2 when sometimes smoking was answered, 3 when often smoking was answered, 4 when almost everyday smoking was answered. In this study, we defined smoking by a smoking status value of ≥2.

##### 2.2.1.3 Depression

We measured the depression symptoms by the Center for Epidemiologic studies Depression Scale (CES-D) developed by Lenore S. Radloff in 1977 (45). The CES-D consists of 20 items rated by a 4-point Likert scale measuring the number of days participants experienced depressive symptoms in the past week: rarely or none of the time (less than 1 day) =0, some or a little of the time (1-2 days) =1, occasionally or a moderate amount of the time (3-4 days) =2, most or all of the time (5-7 days) =3. The total score of the CES-D ranges from 0-60, and the degree of depressive symptoms increased with the score increase during the last week. Besides, 4 in 20 items of the CES-D are reverse-scored. (eg. “I felt I was just as good as other people”, “I felt hopeful about the future”, “I was happy”, “I enjoyed life”.) The CES-D is a widely used scale to assess the depressive symptoms from adolescents to elderly people across different countries and regions in the world (46–48). The Cronbach’s α of the CES-D in this study was 0.887.

##### 2.2.1.4 Data analysis

All data analyses were conducted with IBM SPSS version 25.0 First, descriptive statistics were performed to analyze demographic characteristics of the participants. Categorical variables were described as frequency (n) and percentage (%), and the mean and standard deviation (S.D.) or the median and interquartile range (IQR) was used to describe continuous variables depending on whether its normally distributed. The chi-square test, Students’ t-test or Mann-Whitney U test were used to compare the difference between groups as appropriate. Second, the Spearman correlation analysis was carried out among Internet addiction, school bullying and depression.

Third, we used the Model 4 with bootstrapping 5,000 samples in the PROCESS v4.2 developed by Andrew F. Hayes (www.processmacro.org) for the mediation analysis investigating the mediating effect of depression on the association between Internet addiction and school bullying. In the mediation analysis, the independent variable (X) was school bullying, Internet addiction was used as the dependent variable (Y), mediator variable (M) was depression, and age and gender were control variables.

Finally, Model 7 was used for moderation analysis with bootstrapping 5,000 samples in the P PROCESS v4.2. In the moderation analysis, the independent variable (X) was school bullying, Internet addiction was the dependent variable (Y), mediator variable (M) was depression, smoking status was used as the moderator variable(W), and age and gender were control variables.

All *p* value <0.05 considered as statistically significant.

## 3 Results

### 3.1 Baseline characteristics of the participants

The demographic characteristics of the participants stratified by sex are shown in **Table 1**. Among the participants, 896 (44.98%) were females (15.88 ± 1.604 years) and 1096 (55.02%) were males (15.94 ± 1.648 years). Males had a significantly higher prevalence of smoking (11.9% *vs.* 3.6%, *p* < 0.001), drinking (2.7% *vs.* 1.2%, *p* < 0.05), and school bullying behaviors (31.1% *vs.* 14.2%, *p* < 0.001) than females. However, with respect to Internet addiction, 301 (34.4%) were females and 244 (22.9%) were males (*p* < 0.001). And self-reported depressive symptoms were more serious in females than in males, as indicated by the higher score of the CES-D scale (*p* < 0.001).

**Table 1:** Baseline characteristics of the participants by gender.

Variables	Overall (n=1992)	Male (n=1096)	Female (n=896)	Chi-square/Z
		*N* (%)/(*M* ± *SD*)	*N* (%)/(M ± SD)	
Age	15.92 ± 1.628	15.94 ± 1.648	15.88 ± 1.604	0.786
Smoking Status				45.346***
Yes	162 (8.1%)	130 (11.9%)	32 (3.6%)	
No	1830 (91.9%)	966 (88.1%)	864 (96.4%)	
Drinking Status				5.573*
Yes	41 (2.1%)	30 (2.7%)	11 (1.2%)	
No	1951 (97.9%)	1066 (97.3%)	885 (98.8%)	
Number of friends				7.164*
1 or less	83 (4.2%)	43 (3.9%)	40 (4.5%)	
2 - 3	235 (11.8%)	111 (10.1%)	124 (13.8%)	
More than 3	1674 (84.0%)	942 (85.9%)	732 (81.7%)	
Academic performance				12.717*
very low	144 (7.2%)	82 (7.5%)	62 (6.9%)	
low	347 (17.4%)	213 (19.4%)	134 (15.0%)	
moderate	435 (21.8%)	252 (23.0%)	183 (20.4%)	
high	697 (35.0%)	359 (32.8%)	338 (37.7%)	
very high	369 (18.5%)	190 (17.3%)	179 (20.0%)	
Family affluence				17.730***
Poor	119 (6.0%)	86 (7.8%)	33 (3.7%)	
Average	1467 (73.6%)	777 (70.9%)	690 (77.0%)	
Rich	406 (20.4%)	233 (21.3%)	173 (19.3%)	
Only child				4.916*
Yes	374 (18.8%)	225 (20.5%)	149 (16.6%)	
No	1618 (81.2%)	871 (79.5%)	747 (83.4%)	
Internet addiction				31.484***
Yes	545 (28.0%)	244 (22.9%)	301 (34.4%)	
No	1398 (72.0%)	823 (77.1%)	575 (65.6%)	
School bullying				78.696***
Yes	468 (23.5%)	341 (31.1%)	127 (14.2%)	
No	1524 (76.5%)	755 (68.9%)	769 (85.8%)	
Parent-teen Communication				4.314
very little or no communication	132 (6.6%)	63 (5.7%)	69 (7.7%)	
occasional communication	821 (41.2%)	468 (42.7%)	353 (39.4%)	
regular communication	1039 (52.2%)	565 (51.6%)	474 (52.9%)	
CES-D	39.03 ± 11.462	37.2 ± 10.739	41.27 ± 11.920	-7.920***

1. *** *p* < 0.001, ** *p* < 0.01, * *p* < 0.05;.

2. 49 missing cases of Internet addiction.

### 3.2 Correlation analysis

Regarding the means, standard deviations, and correlations between school bullying, depression, and Internet addiction (**Table 2**), school bullying was positively correlated with depression (*r_s_* =0.308, *p* < 0.01) and Internet addiction (*r_s_* =0.340, *p* < 0.01), and depression was positively correlated with Internet addiction (*r_s_* =0.448, *p* < 0.01). The results showed that a significant correlation existed between school bullying, depression, and Internet addiction.

**Table 2:** Descriptive statistics and correlations between variables.

	Mean	SD	School bullying	Depression	Internet addiction
School bullying	40.51	5.816	1		
Depression	39.03	11.462	0.308**	1	
Internet addiction	42.45	14.218	0.340**	0.448**	1

** *p* < 0.01.

### 3.3 The mediation effect of depression

The mediating effect of depression on the association between school bullying and Internet addiction is shown in **Table 3**. After controlling gender and age, school bullying positively predicted Internet addiction (direct effect) [*β* = 0.565, *SE*= 0.053, 95% *CI* (0.461, 0.669)]. Meanwhile, the mediation model revealed that school bullying positively predicted depression [*β* = 0.760, *SE*= 0.041, 95% *CI* (0.679, 0.841)], and depression positively predicted Internet addiction [*β* = 0.426, *SE*= 0.027, 95% *CI* (0.373, 0.479)]. Furthermore, the total effect of school bullying on Internet addiction was 0.888 [*SE*= 0.052, 95% *CI* (0.786, 0.990)], and the indirect effect of school bullying was 0.324 [*SE*= 0.031, 95% *CI* (0.266, 0.387)], all the bootstrap 95% *CI* were not included 0, indicating that depression partially mediated the association between school bullying and Internet addiction, and the ratio of indirect to the total effect (effect size) was 36.5%.

**Table 3:** The mediating effect of depression on the relationship between school bullying and Internet addiction.

Variables	*R^2^*	*F*	*β*	*S.E.*	*T*	*P*	*95% CI*
Depression (Y)	0.177	138.707***					
School bullying			0.760	0.041	18.401	< 0.001	(0.679, 0.841)
Internet addiction (Y)	0.252	163.209***					
School bullying			0.565	0.053	10.649	< 0.001	(0.461, 0.669)
Depression			0.426	0.027	15.826	< 0.001	(0.373, 0.479)
Total effect	0.155	118.84***	0.888	0.052	17.089	< 0.001	(0.786, 0.990)
Indirect effect			0.324	0.031			(0.266, 0.387)

1. Gender, age as the control variable.

2. *** p < 0.001.

3. 49 missing cases of Internet addiction.

### 3.4 The moderation effect of smoking

**Figure 2:**
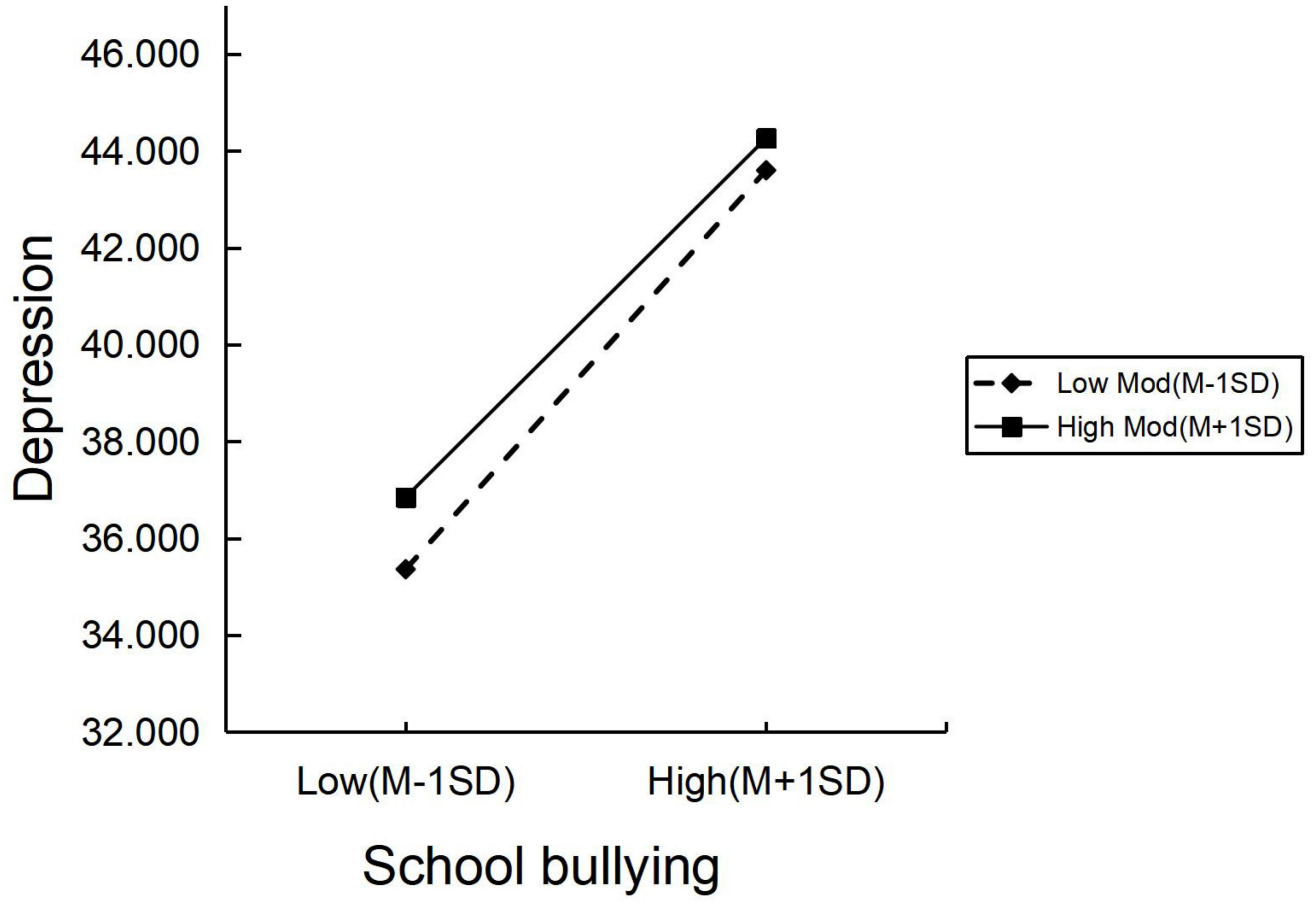
A simple slope test on the moderating effect of smoking.

The moderated mediation analysis displayed in **Table 4** represents that smoking significantly moderated the relationship between school bullying and depression [*β* = -0.166, *SE*= 0.058, 95% *CI* (-0.280, -0.052)]. We also conducted a simple slopes test as shown in **Figure 2**, students with higher levels of school bullying reported higher levels of depression. This relationship between school bullying and depression was stronger when students with a high level (1 SD above mean) of smoking compared to the students with a low level (1 SD below mean) of smoking. Thus, the higher level of smoking was a risk factor when examining the association between school bullying and depression in the students.

**Table 4:** Testing the moderating effect of smoking.

Variables	*R^2^*	*F*	*β*	*S.E.*	*T*	*P*	*95% CI*
Depression (Y)	0.182	85.94***					
School bullying			0.773	0.045	17.320	< 0.001	(0.686, 0.861)
Smoking			2.359	0.772	3.056	0.002	(0.845, 3.872)
School bullying × Smoking			-0.166	0.058	-2.848	0.004	(-0.280, -0.052)
Internet addiction (Y)	0.252	163.209***					
School bullying			0.565	0.053	10.649	< 0.001	(0.461, 0.669)
Depression			0.426	0.027	15.826	< 0.001	(0.373, 0.479)

1. Gender, age as the control variable.

2. *** p < 0.001.

3. 49 missing cases of Internet addiction.

## 4 Discussion

The present study investigated the relationship between school bullying and Internet addiction among southeastern Chinese adolescents, and examined not only the mediation effects of depression symptoms but also the moderation effects of smoking behaviors on the relationship. Our results confirmed the positive association between school bullying and Internet addiction, and supported depression mediating the relationship between school bullying and Internet addiction, and this relationship is strengthened by smoking behaviors.

As expected in hypothesis 1, the degree level of Internet addiction increased with the higher frequency of school bullying behaviors in the adolescents. This finding is consistent with previous studies (11, 49–51). In terms of school bullying victimization predicted adolescents’ Internet addiction behaviors, one popular explanation was that Internet use tends to be a strategy to escape distress, reduce anxiety, and improve mood due to the victimization (52). For example, bullying victims reported feeling better using the Internet (12). On the other hand, school bullying perpetration is also related to Internet addiction. The youth who were bullying perpetrators were more likely to weaken social bonds, have unhealthier relationships, and spend more time online, increasing the risk of developing Internet addiction (53).

Our findings highlight that school bullying has a positive effect on depression symptoms, which sequentially increased the Internet addictive disorders in adolescents. These results proved that depression could be an important mediating variable in the relationship between school bullying and Internet addiction. Prior research has also shown that depression mediated the relationship between school bullying behaviors and Internet addiction in adolescents (31), suggesting that adolescents’ school bullying behaviors could predict depressive symptoms, and depressive behaviors may lead to Internet addiction. According to Beck’s cognitive model of depression, a vulnerable individual developeds depressive symptoms due to environmental triggers (54), and school bullying victimization could be the adverse environmental trigger that led to adolescent’s depression. Besides, the co-occurrence of Internet addiction and depression has also been supported by previous studies (55–57). Thus, taking measures to identify and intervene in adolescents’ depressive symptoms is critical in the prevention of school bullying and Internet addiction.

In line with previous studies (58, 59), our study also suggests that the score of CES-D in girls was higher than in boys, which might point out that there are gender differences when examining the association of school bullying and Internet addiction mediated by depression. The elaborated cognitive vulnerability-transactional stress theory could help explain the mechanism of gender difference from the aspects of negative life events, initial negative affect, and cognitive vulnerability to depression among adolescent girls (60). In this case, gender-specific intervention strategies should be taken into consideration when controlling depressive symptoms or disorders in adolescent girls.

This study further suggests that the relationship between school bullying and Internet addiction through depression is moderated by adolescents’ smoking behaviors, and there was an interaction between school bullying and smoking status in predicting adolescents’ depressive symptoms. Specifically, in those with a higher level of smoking, school bullying behaviors may increase their depressive symptoms, as smoking promotes depression when adolescents experience school bullying. The strong evidence from a Chinese large-scale cross-sectional study concluded that current smoking status was positively associated with depression or school bullying behaviors in girls and school bullying in boys could help explain the important moderating role of smoking when studying school bullying and depression in our study (35). Moreover, both cross-sectional and longitudinal studies have found that adolescents’ smoking initiation and more frequent smoking are associated with hostility and depression (61, 62).

One possible neurobiological mechanism of how smoking status is associated with bullying behaviors could be explained by the fact that nicotine and other components of cigarettes may influence brain regions and neural circuitry of aggression, which in turn increase the risk of psychological issues such as depression (33, 63). On the other hand, the control theory could also be an aspect to understand adolescent’s cigarette smoking behaviors on the relationship between school bullying and depression (64), from which adolescents who are smoking are considered to have low self-regulation, and are more likely to be involved in school bullying events and increase the risk of depression (65). Therefore, identifying cigarette smoking adolescents and making targeted intervention measures on smoking control could be efficient when preventing adolescents’ school bullying and its consequences.

## 5 Limitations

There are several limitations in our study despite the fact that we provided new perspectives for preventing school bullying and Internet addiction through depression and cigarette smoking intervention. First of all, the cross-sectional study design in our study limited the establishment of causality between school bullying, depression,smoking, and Internet addiction. Longitudinal studies are needed to clarify the possible causal relationships between school bullying, depression, smoking, and Internet addiction. Second, the current study included adolescents only from one southeastern Chinese city, which might not be generalized to adolescents from other regions of China. Future studies should be designed as multi-center designs with different cities and regions to verify the representativeness of our findings. In addition, both reporting bias and recall bias could not be neglected in the study since all the data were collected from the self-report questionnaire-based survey, adolescents may underreport or exaggerate when answering. To increase the validity of the findings, comprehensive methods of collecting data such as interviews from parents, teachers or peers instead of only collecting from adolescents themselves could be more reliable (51). Fourth, the smoking status was assessed using a single self-reported item, validated scales or biomarkers should be taken into consideration in future studies to increase the accuracy of smoking. Moreover, it is found that depression partially mediates the relationship between school bullying and Internet addiction among adolescents, other mediators (e.g., socioeconomic status, parental supervision, and peer influence) deserve to be further studied in the future to supplement a more systematic exploration of the relationship between school bullying and Internet addiction among youth. Finally, bidirectional effects between school bullying and Internet addiction, different sub-types of school bullying (e.g., physical victimization and perpetration, verbal victimization and perpetration, relational victimization and perpetration, cyber victimization and perpetration) and gender differences of depression should also be considered to supplement our findings in the future.

## 6 Practical implications

Despite these limitations, our findings have several implications for the prevention of adolescents’ Internet addiction. First, it is important to launch the anti-school bullying program in schools considering the positive correlation between school bullying and Internet addiction among adolescents. Second, the mediation role of depression suggests that mental health courses incorporating the depression intervention module would be effective in an adolescent’s Internet addiction intervention. Third, encouraging adolescents to quit smoking could decrease the risk of developing depression, and prevent Internet addiction consequently.

## 7 Conclusion

In conclusion, this study found a significant mediating effect of depression on the association of school bullying involvement and Internet addiction among Chinese southeastern adolescents, and we also found the moderating effect of smoking on the association between school bullying involvement and depression among Chinese southeastern adolescents. Thus, interventions targeting depression and smoking should be taken into consideration in the prevention of Internet addiction among Chinese southeastern adolescents related to school bullying.

## Data Availability

The original contributions presented in the study are included in the article/supplementary material. Further inquiries can be directed to the corresponding author.
